# Prediction of the Drug–Drug Interaction Types with the Unified Embedding Features from Drug Similarity Networks

**DOI:** 10.3389/fphar.2021.794205

**Published:** 2021-12-20

**Authors:** Xiao-Ying Yan, Peng-Wei Yin, Xiao-Meng Wu, Jia-Xin Han

**Affiliations:** ^1^ College of Computer Science, Xi’an Shiyou University, Xi’an, China; ^2^ School of Electronic Engineering, Xi’an Shiyou University, Xi’an, China

**Keywords:** drug-drug interaction, random walk with restart (RWR), positive pointwise mutual information (PPMI), multi-model deep autoencoder (MDA), deep neural network (DNN)

## Abstract

Drug combination therapies are a promising strategy to overcome drug resistance and improve the efficacy of monotherapy in cancer, and it has been shown to lead to a decrease in dose-related toxicities. Except the synergistic reaction between drugs, some antagonistic drug–drug interactions (DDIs) exist, which is the main cause of adverse drug events. Precisely predicting the type of DDI is important for both drug development and more effective drug combination therapy applications. Recently, numerous text mining– and machine learning–based methods have been developed for predicting DDIs. All these methods implicitly utilize the feature of drugs from diverse drug-related properties. However, how to integrate these features more efficiently and improve the accuracy of classification is still a challenge. In this paper, we proposed a novel method (called NMDADNN) to predict the DDI types by integrating five drug-related heterogeneous information sources to extract the unified drug mapping features. NMDADNN first constructs the similarity networks by using the Jaccard coefficient and then implements random walk with restart algorithm and positive pointwise mutual information for extracting the topological similarities. After that, five network-based similarities are unified by using a multimodel deep autoencoder. Finally, NMDADNN implements the deep neural network (DNN) on the unified drug feature to infer the types of DDIs. In comparison with other recent state-of-the-art DNN-based methods, NMDADNN achieves the best results in terms of accuracy, area under the precision-recall curve, area under the ROC curve, F1 score, precision and recall. In addition, many of the promising types of drug–drug pairs predicted by NMDADNN are also confirmed by using the interactions checker tool. These results demonstrate the effectiveness of our NMDADNN method, indicating that NMDADNN has the great potential for predicting DDI types.

## Introduction

Combined drug therapies are becoming the prevalent approach for complex disease in recent years, especially for elders who suffer from multiple diseases, such as hypertension, hyperlipidemia, cardiopathy, and cancers (Foucquier and Guedj, 2015; Li et al., 2015; MadaniTonekaboni et al., 2018). Taking two or more medications simultaneously can make use of the complementarity of drug efficacy to treat diseases better. However, drug–drug interactions (DDIs) may cause adverse drug events (ADEs), reduce the efficacy, and so on. Generally speaking, the reaction mode of DDIs can be divided into three categories: synergistic, antagonistic, and no reaction (Sun et al., 2018). The synergistic reaction is the best result for combined drug therapies, meaning that the efficacy of drug A&B is bigger than the sum of drug A efficacy and drug B efficacy. The antagonistic reaction is the worst result for combined drug therapies, which results in reduced efficacy, and the efficacy of drug A&B is smaller than the sum of drug A efficacy and drug B efficacy. Even worse, an antagonistic reaction may lead to toxicity and other adverse effects, which could threaten patients’ lives. The type of no reaction is that the efficacy of drug A&B is equal to the sum of drug A efficacy and drug B efficacy; that is, there is no interaction between drugs A and B. Antagonistic DDIs are associated with 30% of all reported ADEs (Edwards and Aronson, 2000; Tatonetti et al., 2012). Therefore, identifying the types of DDIs is very important in drug research, and it is helpful for safer and effective drug combined prescriptions, and also may help in understanding the causes of side effects of existing drugs.

The types of DDIs can be identified by biochemical experimental (or *in vivo*) methods, but experimental methods are usually time-consuming, tedious and expensive and sometimes lack reproducibility (Gao et al., 2015; Fang et al., 2017). Thus, it is highly desired to develop computational methods (or *in silico*) for efficiently and effectively analyzing and detecting new DDI pairs, and a variety of theoretical and computational methods have been developed to predict DDI types in recent years (Herrero-Zazo et al., 2013; Cheng and Zhao, 2014; Gottlieb et al., 2014; Zhang et al., 2015; Liu et al., 2016; Takeda et al., 2017; Zhang et al., 2017a; Zhang et al., 2017b; Andrej et al., 2018; Ryu et al., 2018; Yu et al., 2018; Lee et al., 2019; Deng et al., 2020; Feng et al., 2020; Harada et al., 2020; Lin et al., 2020; Fatehifar and Karshenas, 2021; Wang et al., 2021). Computational methods can guide experimentalists designing the best experimental scheme, narrowing the scope of candidate DDIs, and provide supporting evidence for their experimental results.

Generally, the computational methods for DDI prediction include two scenarios: One is predicting whether two drugs interact or not, and the other is predicting which type of interactions, events or effects exist between two drugs. Essentially, the former can be viewed as a binary classification problem, whereas the latter is a multiclassification problem. Both can be used for better understanding of drugs, especially for explaining the occurrence of ADEs. For example, DPDDI (Feng et al., 2020) combines a GCN-based feature extractor and deep neural network (DNN)-based predictor to predict whether two drugs are interacted or not. DeepDDI (Ryu et al., 2018) uses the structures of chemical compounds to predict 86 DDI types.

Usually, the existing computational method for predicting DDI-associated types can be classified into two categories: text mining– and machine learning–based methods. The text mining–based methods are mainly for tackling DDI prediction as a task of identifying the semantic relation between the two drugs in natural language processing (NLP) from public corpora or biomedical texts (Liu et al., 2016; Zhang et al., 2017a; Fatehifar and Karshenas, 2021). They are very useful in building DDI-related databases. For example, Herrero-Zazo (Herrero-Zazo et al., 2013) built a manually annotated corpus for DDIs in biomedical texts, which are obtained from 730 DrugBank documents and 175 MEDLINE abstracts and annotated DDI relationships into four types: *mechanism* (when the pharmacokinetic mechanism of a DDI is described), *effect* (when the effect of a DDI is described), *advice* (when recommendation or advice regarding a DDI is given), and *int* (when sentence simply states that a DDI occur and does not provide any information about the DDI). Based on these data sets, Zhang et al. (Liu et al., 2016) presents a DDI extraction method by hierarchical RNNs on sequence and shortest dependency paths. However, the performance of text mining–based methods is affected by the quality and the amount of the training data, and the text mining–based methods cannot find new DDIs beyond the texts. These methods cannot give suggestions to doctors before a combinational treatment is made (Takeda et al., 2017). In contrast, machine learning–based methods provide a promising way to identify unannotated potential DDIs for downstream experimental validations.

Prior machine learning–based methods apply KNN (Andrej et al., 2018), SVM (Andrej et al., 2018), logistic regression (Cheng and Zhao, 2014; Gottlieb et al., 2014; Takeda et al., 2017), decision tree (Cheng and Zhao, 2014), naïve Bayes (Cheng and Zhao, 2014), and network-based label propagation (Zhang et al., 2015) and random walk (Zhang et al., 2017b) or matrix factorization (Yu et al., 2018) to detect DDIs. These methods are based on drug properties, such as chemical structure (Cheng and Zhao, 2014; Gottlieb et al., 2014; Zhang et al., 2015; Zhang et al., 2017b; Andrej et al., 2018), targets (Cheng and Zhao, 2014; Gottlieb et al., 2014; Takeda et al., 2017), Anatomical Therapeutic Chemical classification (ATC) codes (Cheng and Zhao, 2014; Gottlieb et al., 2014; Andrej et al., 2018), side effects (Gottlieb et al., 2014; Zhang et al., 2017b; Yu et al., 2018), et al. Most of these studies are based on one or several of the abovementioned off-the-shelf features of drugs or the tailored similarity functions, such as kernel functions.

In recent years, deep learning is becoming a promising technique for automatically capturing chemical compound features from data sets, and it successfully improves predictive performance. For example, Harada et al. (2020) constructed a dual graph convolutional neural network to predict DDIs by combining the internal and external graph structures of drugs to learn low-dimensional representations of compounds. However, this method works well only for moderately dense chemical networks with heavy-tailed degree distributions. Wang et al. (2021) combined interview information of drug molecular and intraview of DDI relationships, developing a graph contrastive learning framework to predict DDIs. Lin et al. (2020) merged several data sets into a vast knowledge graph with 1.2 billion triples, constructing KGNN to resolve the DDI prediction. On the other side, based on the structural, gene ontology term, and target gene similarity profiles, Lee et al. (2019) applied an autoencoder to reduce the dimensions of each profile, constructing a DNN model by combining all the reduced features to predict the types of DDIs. Deng et al. (2020) used the chemical substructures, targets, enzymes, and pathways of drugs to compute a similarity matrix of drugs, inputting each matrix to a DNN model, and combining the four submodels to predict DDI events. Besides DDI prediction, deep learning is also successfully applied for drug–target interaction prediction; for example, Shang et al. (2021) develop a multilayer network representation learning method to learn the feature vectors of drugs and target. An et al. (An and Yu, 2021) use biased RWR and Word2vec algorithms to obtain the feature representation of drugs and targets.

Although the above works have made crucial efforts on DDIs and the types of DDI prediction, there still exists space for improvement. First, the methods developed so far are mostly to integrate one or more features directly, but not capture the network structural information of the feature information. Second, a variety of drug features can be obtained from DrugBank (Knox et al., 2010) data sets; however, methods always directly merge different feature vectors or combine the results of each model. Third, the classification accuracy needs to be increased. How to effectively combine more features of a drug is a challenge. In this work, we proposed a unified feature-embedding method for the type of DDI prediction, First, DDI types and drug features were extracted from DrugBank (Knox et al., 2010) data sets, and the Jaccard coefficient was used to construct the similarity networks. Second, random walk with restart algorithm and positive pointwise mutual information were implemented to adjust the drug similarity matrices by capturing network structural information of drug networks. Third, a multimodal deep autoencoder (MDA) was adopted to integrate the heterogeneous information of drugs. Finally, a DNN was built to predict the types of DDIs.

## Data sets

To facilitate benchmarking comparison with other state-of-the-art methods, we used the DDI data sets provided by Deng et al. (2020). The DDI or type descriptions are collected from Drugbank (Knox et al., 2010), which are formalized into a four-tuple structure by using the StanfordNLP tool (Zeman et al., 2018) as drug A, drug B, mechanism, action. For example, the description of “the risk or severity of adverse effects of Abemaciclib can be increased when it is combined with drug Amiodarone” is recorded as (Abemaciclib, Amiodarone, risk or severity of adverse effects, increased); here, the “Abemaciclib” and “Amiodarone” are the names of the two drugs, the “risk or severity of adverse effects” means the effect of drugs “Abemaciclib” and “Amiodarone,” and “action” represents the increase or decrease after combining two drugs. “Mechanism” and “action” are combined to represent the drug interaction type. After removing DDIs associated with more than one interaction type and also removing the interaction types with fewer than 10 DDIs, finally, the DDI data set contains 572 drugs, 74,528 pairs of DDIs, and 65 types of interactions. The data set is available at https://github.com/YifanDengWHU/DDIMDL/event.db.

The drug-related heterogeneous features used in this work involve the drug structure information, drug–target association data set, drug–enzyme association data set, drug–pathway association data set, and ATC code of drugs. All of these are extracted from the Drugbank database (Version 3.0) (Knox et al., 2010).

## Methods

**FIGURE 1:**
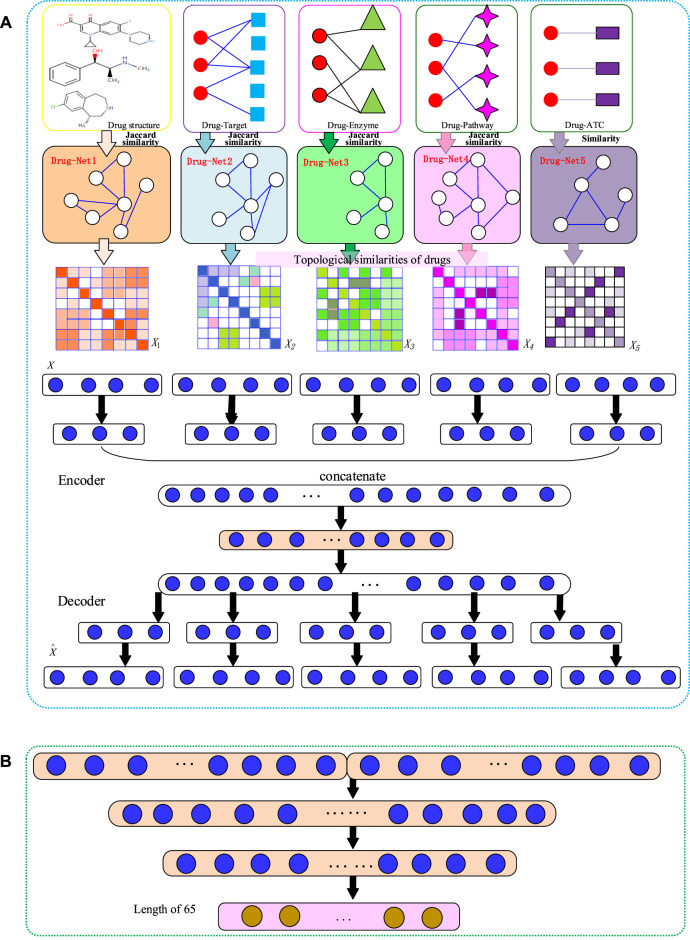
The flowchart of NMDADNN. **(A)** The network-integrated MDA feature extractor with three steps: 1) computing drug similarity matrices; 2) generating drug topological similarity networks by using RWR and PPMI; 3) integrating these network-based similarity matrices with the MDA method to form the unified embedding feature description of drug. **(B)** The DNN-based predictor.

Our NMDADNN method can be divided into four parts: 1) extracting drug features and computing similarity between drugs, 2) adjusting the drug similarity matrices by using random walk with restart algorithm and to compute positive pointwise mutual information for capturing the network structural information, 3) integrating the five drug similarity matrices with the MDA method to obtain the unified embedding features for representing each drug, and 4) constructing the drug–drug pair $\left\{d_{i},d_{j}\right\}$ features by concatenating the unified embedding features of drug $d_{i}$ and drug $d_{j}$ and feeding them into the DNN to predict the type of DDI interaction. The flowchart of the NMDADNN method is shown in Figure 1.

### Generating Drug Similarity Matrices

The drug-related heterogeneous features involve the chemical substructure information, drug–target association, drug–enzyme association, drug–pathway association, and ATC code of drugs. Each feature corresponds to a set of feature descriptors. The PubChem fingerprint list consisting of 881 chemical substructures (Nair and Hinton, 2010) is used to encode drug chemical structures. Formally, drug $d_{i}$ is defined as a *w*-dimensional binary vector: $f_{d_{i}}^{struct}=\left[s_{i1},\cdots ,s_{il},\cdots ,s_{iw}\right]$ , where *w* = 881, $s_{il}=1$ if the *l*th substructure in the PubChem fingerprint list exists in drug $d_{i}$ ; otherwise, $s_{il}=0$ . As extracted from the Drugbank database (Version 3.0) (Knox et al., 2010), there are 1162 types of targets associated with drugs; thus, the target feature can be defined as $f_{d_{i}}^{t\arg et}=\left[t_{i1},\cdots ,t_{il},\cdots ,t_{iq}\right]$ , where *q* = 1162, $t_{il}=1$ if the *l*th target is associated with drug $d_{i}$ ; otherwise, $t_{il}=0$ . Similarly, as there are 957 pathways associated with drugs, the pathway feature can be defined as $f_{d_{i}}^{pathway}=\left[p_{i1},\cdots ,p_{il},\cdots ,p_{ik}\right]$ , where *k* = 957, $p_{il}=1$ if the *l*th pathway is associated with drug $d_{i}$ ; otherwise, $p_{il}=0$ . There are 202 enzymes associated with drugs, and the enzymes feature can be defined as $f_{d_{i}}^{enzyme}=\left[e_{i1},\cdots ,e_{il},\cdots ,e_{ih}\right]$ , where *h* = 202, $e_{il}=1$ if the *l*th pathway is associated with drug $d_{i}$ ; otherwise, $e_{il}=0$ . These features are high dimensional and sparse.

We use the Jaccard similarity coefficient to measure the similarity between drugs *i* and *j* as follows:

$$s\left(d_{i},d_{j}\right)=\frac{\left|f_{d_{i}}\cap f_{d_{j}}\right|}{\left|f_{d_{i}}\cup f_{d_{j}}\right|}$$ (1)

As the ATC classification provides hierarchically semantic codes for drugs (Srivastava et al., 2014) (e.g., the ATC code B01AC06). The ATC-based similarity between two drugs is computed by counting the common subcodes from top to bottom in the hierarchy. For example, an ATC code represented by a vector consists of *N* entries, and each entry denotes the subcode in its corresponding level of ATC hierarchy. If the first *k* entries in two vectors are the same, the ATC similarity between the two drugs is sATC(di,dj)=kN . We generate five drug similarity matrices, denoted as $S^{struct}={\left(s_{ij}^{ss}\right)}_{m\times m}$ , $S^{t\arg et}={\left(s_{ij}^{tt}\right)}_{m\times m}$ , $S^{pathway}={\left(s_{ij}^{pp}\right)}_{m\times m}$ , $S^{enzyme}={\left(s_{ij}^{ee}\right)}_{m\times m}$ , and $S^{ATC}={\left(s_{ij}^{ATC}\right)}_{m\times m}$ .

### Generating Drug–Drug Similarity Networks

Instead of directly fusing five similarity matrices/network information (i.e., $S^{struct}$ , $S^{t\arg et}$ , $S^{pathway}$ , $S^{enzymes}$ , and $S^{ATC}$ ), we capture the topological structural information of each similarity network by implementing the RWR algorithm on $S^{struct}$ , $S^{t\arg et}$ , $S^{pathway}$ , $S^{enzymes}$ , and $S^{ATC}$ to obtain the feature vectors of the drugs. As fewer hyperparameters and lower computation are needed in the RWR algorithm, it has been widely applied in the complex network analyzing and feature representation learning (Yan et al., 2016; Luo et al., 2017). The RWR algorithm can be formulated as

$$p^{t}=\alpha p^{t-1}W+\left(1-\alpha \right)p^{0},\alpha \in \left(0,1\right)$$ (2)

where $p^{t}$ is a *m*-dimensional vector in which the *i*th element represents the label confidence score of drug $d_{i}$ at time step *t*, and $p^{0}$ is a *m*-dimensional initial one-hot vector with the value of the *i*th entry being 1 and all other entries being 0; similarity matrix *S* is normalized as $W=D^{-1}*S$ , where *D* is the diagonal degree matrix with $D_{ii}=\sum_{j}S_{ij}$ , and *α* is restart probability controlling the relative influence of local and global topological information. When the L1 norm of $\Delta p=p^{t}-p^{t-1}$ is less than a small positive ε (here, we set ε = 10^−9^), we can obtain a stationary distribution vector *p*, which is referred to as the diffusion state of each node (Cho et al., 2015), and all the distribution vectors *p* of drugs are organized as matrix *p*.

Then, we calculate the topological similarity of each node by using PPMI, which contains rich network context information and is defined as

$$X\left(i,j\right)=\max\left(0,\log_{2}\frac{P\left(i,j\right)*\sum_{i}\sum_{j}P\left(i,j\right)}{\sum_{i}P\left(i,j\right)*\sum_{j}P\left(i,j\right)}\right)$$ (3)

The matrix *X* is a nonsymmetric matrix. We use the average of X(i,j) and X(j,i) to represent the topological similarity of drugs *i* and *j*, denoted as *N1, N2*, *N3*, *N4*, and *N5.*

### Generating the Unified Embedding Feature Vectors of Drugs with MDA

After obtaining the five drug topological similarity matrices *N1, N2*, *N3*, *N4*, and *N5*, we generate the unified embedding feature vectors with MDA (Zeng et al., 2019) to represent each drug. MDA can integrate multiple PPMI matrices by nonlinear mapping of all the similarity matrices $N_{j}\in R^{m\times m}$ into a unified embedding feature space $H_{\text{n}}\in R^{d_{n}*m}$ . Following the standard definition of autoencoder (Vincent et al., 2010), we formulate the process of MDA in the following sections:

#### Encoder

First, we map each network $N_{j}\left(j=1,...5\right)$ into a low-dimensional nonlinear embedding:

$$H_{\text{encode}}^{\left(j\right)}=\sigma \left(W_{encode}^{\left(j\right)}N^{\left(j\right)}+B_{encode}^{\left(j\right)}\right)$$ (4)

where $W_{encode}^{\left(j\right)}\in R^{d_{j}\times m}$ is the weight matrix and $B_{encode}^{\left(j\right)}\in R^{d_{j}\times m}$ is the bias matrix, respectively, and $\sigma \left(x\right)=\frac{1}{1+e^{-x}}$ is the sigmoid activation function.

Then, we concatenate the low-dimensional embedding features obtained above as $H=\left[H_{encode}^{\left(1\right)},...,H_{encode}^{\left(5\right)}\right]$ and apply multiple nonlinear functions on *H* as

$$H_{c,1}=\sigma \left(W_{1}H+B_{1}\right)$$ (5)

$$H_{c,l+1}=\sigma \left(W_{l}H_{c,l}+B_{l}\right)$$ (6)

where l∈{1,...,L} is the number of layers for the successive integrated embedding and $H_{c,L}$ is the optimal unified common layer.

#### Decoder

We first reconstruct the drug representation $H_{c,2L}$ from the last encoding unified embedding layer $H_{c,L}$ . The layer number in the decoder is equal to the layer number *L* in the encoder, and then we compute the individual representation $H_{\text{decode}}^{\left(j\right)}$ from the reconstructed drug representation $H_{c,2L}$ ,

$$H_{\text{decode}}^{\left(j\right)}=\sigma \left(W_{decode,1}^{\left(j\right)}H_{c,2L}+B_{decode,1}^{\left(j\right)}\right),\;j=1,...5$$ (7)

Finally, we reconstruct PPMI matrices $N_{j}\left(j=1,...5\right)$ by mapping $H_{\text{decode}}^{\left(j\right)}$ to the original space as follows:

$${\overset{\wedge }{N}}^{\left(j\right)}=\sigma \left(W_{decode,2}^{\left(j\right)}H_{decode}^{\left(j\right)}+B_{decode,2}^{\left(j\right)}\right)$$ (8)

All the parameters $\theta =\left\{W_{encode}^{\left(j\right)},B_{encode}^{\left(j\right)},W_{decode}^{\left(j\right)},B_{decode}^{\left(j\right)},W_{l},B_{l}\right\}$ for l∈{1,...,2L} are optimized by minimizing the following reconstruction loss between the original and reconstructed PPMI matrix.

$$\underset{\theta }{\arg\min}\sum_{j=1}^{5}Loss\left(N^{\left(j\right)},{\overset{\wedge }{N}}^{\left(j\right)}\right)$$ (9)

Here, the input layer of the MDA encoder includes five networks, and these networks have 572 features for each drug. The input layer of the MDA maps 572 features to 256 embedding features. The concatenate layer of the MDA maps 256×5 to 640 unified common features.

### Predicting DDI Types with DNN

We build the following DNN to predict DDI types.

$$\mathrm{Pr}\left(y|X,\theta \right)=f\left(Z_{out}W_{out}+b_{out}\right)$$ (10)

$$Z_{out}=\sigma \left(Z_{k}W_{k}+b_{k}\right)$$ (11)

where $X\in {ℜ}^{n\times p}$ is the input feature matrix with *n* samples and *p* features and $y\in {ℜ}^{n\times t}$ is the predicted labels of DDIs. θ denotes all the parameters involved in the model, $Z_{out}$ and $Z_{k}\left(k=1,\cdots l\right)$ are the hidden neurons with corresponding weight matrices $W_{out}$ and $W_{k}$ , $b_{out}$ and $b_{k}$ are the bias vectors. σ(⋅) is the activation function, such as sigmoid, hyperbolic tangent (tanh), rectifiers or rectified linear unit (ReLU). f(⋅) is the softmax activation function, which is used in the last layer to convert values of the output layer into probability predictions.

In this work, we combine the unified embedding features for each drug–drug pair as $X=\left[H_{c,L}^{d_{i}},H_{c,L}^{d_{j}}\right]$ and feed X into the DNN model. Adam optimizer (Kingma and Ba, 2015) is used to train the model, and ReLU (Nair and Hinton, 2010) is used as the activation function. We add batch normalization layers (Ioffe and Szegedy, 2015) to accelerate the convergence and dropout layers (Srivastava et al., 2014) to avoid overfitting. The cross-entropy is chosen as the loss function to optimize the NMDADNN model, and the early stopping strategy (Prechelt, 1998) is adopted to stop the training process.

## Results and Discussion

In this work, we first introduce six metrics and cross-validation test approaches to evaluate the performance of predictors and then compare the performance of NMDADNN with other existing state-of-the-art DNN-based methods on the same data set, discussing the effect of ATC feature, representation strategies, feature aggregate operators, and parameter setting. In the end, we conduct case studies to analyze the potential DDI pairs predicted by NMDADNN and to confirm the usefulness of our NMDADNN method.

### Performance Evaluations

Here, we focus on three kinds of scenes, S1: the prediction of unobserved or potential DDI interaction types between known drugs; S2: the DDI type prediction between known drugs and new drugs; S3: the DDI type prediction between new drugs. The fivefold cross-validation (5-CV) test approach (Yan et al., 2016; Luo et al., 2017) is used to assess the power of predictors in three scenes. For S1, we randomly split all DDI pairs based on the DDI types into five nonoverlapping subsets. In each round of CV, the model is trained on the training set, and the testing set is used for prediction. The procedure repeats five times until all the DDI pairs are tested in turn. For scenes of S2 and S3, the 5-CV is applied for drugs. We randomly split all drugs into five nonoverlapping subsets with roughly equal size, and one set of drugs is removed as the testing set. The other four sets of drugs are referred to as the training set. For S2, the model is trained on the DDI types between the training and training drugs and then making the prediction of DDI types between training and testing drugs. For S3, the model is trained also on the DDI types between training drugs but making the prediction of DDIs types between testing drugs and testing drugs. S2 and S3 are more compatible with the real application cases, in which S2 aims to predict DDI types for new drugs on existing drugs, and S3 aims to predict DDIs types among new drugs.

The final performance in prediction models is measured by the metrics of accuracy (ACC), area under the precision-recall-curve (AUPR), area under the ROC curve (AUC), F1 score, precision and recall. These metrics are defined as follows:

$$ACC=\frac{1}{l}\sum_{i=1}^{l}\frac{TP_{i}+TN_{i}}{TP_{i}+TN_{i}+FP_{i}+FN_{i}}$$ (12)

$$Macro\;recall=\frac{1}{l}\sum_{i=1}^{l}\frac{TP_{i}}{TP_{i}+FN_{i}}$$ (13)

$$Micro\;recall=\frac{\sum_{i=1}^{l}TP_{i}}{\sum_{i=1}^{l}TP_{i}+\sum_{i=1}^{l}FN_{i}}$$ (14)

$$Macro\;precision=\frac{1}{l}\sum_{i=1}^{l}\frac{TP_{i}}{TP_{i}+FP_{i}}$$ (15)

$$Micro\;recall=\frac{\sum_{i=1}^{l}TP_{i}}{\sum_{i=1}^{l}TP_{i}+\sum_{i=1}^{l}FP_{i}}$$ (16)

$$\text{F}1=\frac{2*precision*recall}{precision+recall}$$ (17)

Here, *l* indicates the number of DDI types. We use micrometrics for AUPR and AUC, whereas we use macrometrics for precision, recall, F1, and ACC.

### Comparison of NMDADNN with Other DNN-Based Methods

**FIGURE 2:**
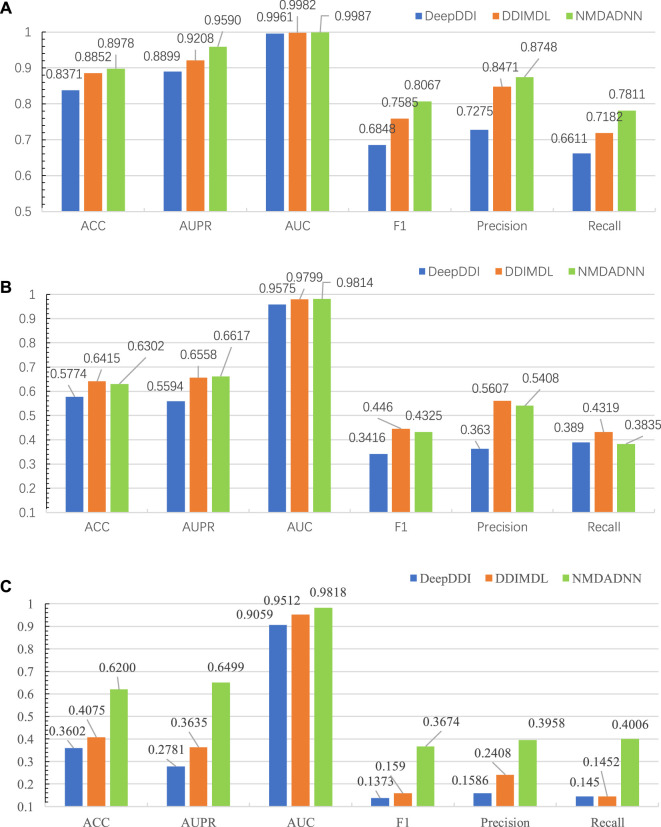
Results of NMDADNN, DDIMDL, and DeepDDI in the 5-CV test. **(A)** S1 scene, **(B)** S2 scene, **(C)** S3 scene.

We compared our NMDADNN method with two other DNN-based methods of DDIMDL (Deng et al., 2020) and DeepDDI (Ryu et al., 2018) in the 5-CV test. DDIMDL predicted DDIs by integrating four DNN-based submodels with the chemical substructures, targets, enzymes, and pathways information of drugs. DeepDDI used the names of drug–drug or drug–food constituent pairs and their structural information as input and adopted DNN to predict DDI type. For S1 scenes, the prediction results of NMDADNN, DDIMDL and DeepDDI on the same data set are shown in Figure 2A, from which we can see that the performance of NMDADNN is superior to the other two methods. For example, ACC, AUPR, AUC, F1 score, precision and recall metrics of NMDADNN are 6.1%, 6.9%, 0.3%, 12.2%, 14.7%, and 12.0% higher than that of DeepDDI and 1.3%, 3.8%, 0.05%, 4.8%, 2.8%, and 6.3% higher than that of DDIMDL, respectively. Moreover, we also evaluated the performances in scenes of S2 and S3, and the prediction results of NMDADNN, DDIMDL, and DeepDDI are shown in Figures 2B,C, respectively. From Figures 2B,C, we can see that all the metrics of the three methods in S2 and S3 are lower than S1, but the performance of NMDADNN is also better than that of DDIMDL and DeepDDI in S2 and S3. These experimental results demonstrate that the NMDADNN method outperforms DeepDDI and DDIMDL for S2 and S3 scenes, which corroborates the efficiency of network-based unified drug representations again. These results show that our NMDADNN can effectively predict the type of DDI, especially for the prediction of interactions between new drugs (S3 scene). More results are provided in the Supplementary Tables S1, S2.

### Effect of Feature Representation Strategies from Multiple Data Resources

To evaluate the effect of different strategies in the process of feature representation, that is, using the RWR algorithm on each similarity network to capture network topological structural features, constructing the PPMI matrix to capture the structure information of the network, and further applying MDA on all the PPMI matrices to obtain a unified, low-dimensional feature representation of drugs, we also designed three approaches of DNN, MDADNN, and NMDADNN. For DNN, we averaged the five drug similarity matrices of $S^{struct}$ , $S^{t\arg et}$ , $S^{pathway}$ , $S^{enzymes}$ , and $S^{ATC}$ to form one integration drug similarity matrix and used the DNN model to predict the types of DDIs. For MDADNN, we used MDA to integrate the five drug similarity matrices of $S^{struct}$ , $S^{t\arg et}$ , $S^{pathway}$ , $S^{enzymes}$ , and $S^{ATC}$ to form one unified common drug representation and adopted the DNN model to predict the potential DDIs. For NMDADNN, before using MDA, we used the RWR algorithm and computed PPMI for capturing the network topological structure matrices (i.e., N1, N2, N3, and N4) of the original similarity matrices. The results of DNN, MDADNN, and NMDADNN in the 5-CV test are shown in Table 1. From Table 1, we can see that all the evaluation metrics of MDADNN are higher than that of DNN, indicating that the unified drug embedding generated with the MDA can improve the predictive performance. Comparing MDADNN with NMDADNN, the precision and recall of NMDADNN are 1.6% and 0.9% higher than that of MDADNN, respectively, meaning that the trick of using the network topological similarity can effectively enhance the power of the predictive method. Results in Table 1 demonstrate the effectiveness of the unified embedding and network-based strategies used in our NMDADNN method.

**TABLE 1:** Results of DDI, MDADDI, and NMDADDI for S1 scene in 5-CV test.

Performance	DNN	MDADNN	NMDADNN
ACC	0.8253	0.8935	0.8978
AUPR	0.8960	0.9549	0.9590
AUC	0.9971	0.9986	0.9987
F1-score	0.6383	0.8034	0.8067
Precision	0.7608	0.8585	0.8748
Recall	0.5820	0.7725	0.7811

### Effectiveness of New Similarity Metrics

To validate whether our ATC-based drug similarity matrix can improve DDI prediction or not, we used five sets of drug similarities (i.e., $S^{struct}$ , $S^{t\arg et}$ , $S^{pathway}$ , $S^{enzymes}$ , and $S^{ATC}$ ) in our NMDADNN method, namely, as NMDADNN_a, and also inputted four sets of drug similarities (i.e., $S^{struct}$ , $S^{t\arg et}$ , $S^{pathway}$ , $S^{enzymes}$ , and $S^{ATC}$ ) to NMDADNN, namely, as NMDADNN_na. The results of NMDADNN_a and NMDADNN_na for S1 in the 5-CV test are listed in Table 2, from which we can see that the ATC-based drug similarity feature can improve the performance. Although NMDADNN_a has a similar F1 score and recall as NMDADNN_na, NMDADNN_a outperforms NMDADNN_na in terms of ACC, AUPR, AUC, and precision.

**TABLE 2:** Results of NMDADNN_a and NMDADDI_na for S1 scene in 5-CV test.

Performance	NMDADNN_na	NMDADNN_a
ACC	0.8935	0.8978
AUPR	0.9562	0.9590
AUC	0.9987	0.9987
F1	0.8088	0.8067
Precision	0.8623	0.8748
Recall	0.7813	0.7811

### Effect of Feature Aggregate Operators

With the obtained unified embedding features for each drug, we used three feature operators, i.e., inner product $X^{1}=H_{c,L}^{d_{i}}⊙H_{c,L}^{d_{j}}$ , summation $X^{2}=H_{c,L}^{d_{i}}⊕H_{c,L}^{d_{j}}$ , and concatenation $X^{3}=\left[H_{c,L}^{d_{i}},H_{c,L}^{d_{j}}\right]$ to combine the drug features of the drug–drug pair into one feature vector and feed $X^{j},j=1,2,3$ into the DNN model, respectively. The results of NMDADNN with different feature aggregate operators for S1 in the 5-CV test are listed in Table 3, from which we can see that the concatenation and the inner product operators achieve better results than summation. In this work, we use the concatenate operator to aggregate the feature vectors of drugs for NMDADNN.

**TABLE 3:** Results of three feature aggregate operators in NMDADDI for S1 scene in 5-CV test.

Operators	Inner product	Summation	Concatenation
ACC	0.8994	0.8276	0.8978
AUPR	0.9607	0.8917	0.9590
AUC	0.9989	0.9972	0.9987
F1-score	0.8089	0.7209	0.8067
Precision	0.8677	0.7578	0.8748
Recall	0.7805	0.7079	0.7811

### Effect of Parameter Settings

The parameters in our NMDADNN could affect the prediction performances. Both the network-integrated MDA feature extractor and the DNN-based predictor need to tune the values of restart probability *α* in RWR, the learning rate, epochs, batch size, dropout rate, and neuro numbers (dimensions) in hidden layers.

Some hyperparameter training algorithm, such as Bayesian optimization, can be used to tune these hyperparameters. In this work, we use a grid search in a feasible hyperparameter space to study the effect of each parameter.

Specifically, for the network-integrated MDA feature extractor, we tuned the value of the epochs from {60, 80, 100, 120}, learning rate (lr) from the list of {0.001, 0.005, 0.01, 0.05, 0.1}, the dropout from {0, 0.01, 0.05, 0.1, 0.2}, and the batch size (B-size) from {32, 64, 128, 256, 512}. The hidden layer (H-dim) for the MDA algorithm includes two parts. The first hidden layer maps each network to a low-dimensional nonlinear embedding, and the other is from the concatenation information of all layers in the first part. For the first part, the neuro number from {256}(1 layer),{256,128}(2-layers), the second part, the neuro number from {[256*5,640](1 layers), [256*5, 640, 320](2 layers), [128*5,320](1 layers), [128*5,320,160](2 layers)}. The DNN-based predictor tuned the epochs from {80, 100, 200, 300, 500}, the learning rate (lr) from{0.0001, 0.001, 0.01, 0.1}, the dropout from {0.1, 0.2, 0.3, 0.5}, the batch size (B-size) from {32, 64, 128, 256, 512}, the hidden layer dimensions (H-dim) is tuned from {[640,320,160](4 layers) [640,320](3 layers) [320, 160](3 layers)}. The optimal hyperparameter values used in this work are shown in Table 4. The restart probability *α* in RWR is a diffusion parameter, which adjusts the relative amount of the information from the initial label information to its neighbors. By tuning *α* from the list of {0.5, 0.6, 0.7, 0.8, 0.9}, we fixed α=0.8 in this work.

**TABLE 4:** The optimal values of parameters in NMDADNN.

Parameters	lr	Epoch	Dropout	B-size	^a^I-dim	H-dim	^b^O-dim
Feature extractor	0.01	80	0	64	572*5	(256*5,640,256*5)	640
predictor	0.001	100	0.2	128	640*2	(640,320,160)	65

a I-dim.

b O-dim denote the neuro numbers in input layer and output layer, respectively.

### Case Studies

To evaluate the power of our NMDADNN in predicting the unobserved types of DDIs, in this section, we designed the experiment similar to the literature (Deng et al., 2020) and used all the DDIs and their types in our data set that were extracted from DrugBank (Knox et al., 2010) to train the prediction model and then predicting the possible interaction types among drugs, which are not annotated to each other in the original DDI network. In the current data set, there are 37,264 labeled DDIs and 126,328 unlabeled drug pairs that involve among 572 drugs. We focused on 10 interaction types, which have the highest frequency numbers from #1 to #10. According to the prediction scores in descending order, we checked the top 20 prediction results that are related to each type and also manually checked whether they have the interactions with the checker tool (https://www.drugs.com/). In the top 20 potential DDIs with higher scores for each interaction type, we found that many of them can be supported the results are listed in Table 5. For example, the interaction between Bexarotene and Modafinil is predicted to cause the type #1 interaction, meaning that the metabolism can be decreased when Bexarotene is combined with Modafinil. According to drugs.com, the evidence shows that Bexarotene may reduce the blood levels of Modafinil, which may make the medication less effective in some cases. On the other side, the interaction between Desmopressin and Maprotiline is predicted to cause the type #2 interaction, meaning that the risk or severity of adverse effects can be increased when Desmopressin is combined with Maprotiline. According to drugs.com, the evidence shows that using Desmopressin together with Maprotiline may increase the risk of developing water retention and a condition known as hyponatremia, which is caused by an abnormal decrease in blood sodium concentration. In severe cases, hyponatremia can lead to seizures, coma, and even death. More evidence about confirmed DDI types is provided in Supplementary Table S3.

**TABLE 5:** The confirmed DDIs and their associated types.

Interaction type	DrugBank IDs	Drug names
#1	DB00307, DB00745	Bexarotene, Modafinil
#2	DB00934, DB00035	Maprotiline, Desmopressin
#3	DB08820, DB01204	Ivacaftor, Mitoxantrone
#4	DB00648, DB06413	Mitotane, Armodafinil
#5	DB00704, DB00459	Naltrexone, Acitretin
#6	DB00366, DB09061	Doxylamine, Cannabidiol
#7	DB00537, DB00969	Ciprofloxacin, Alosetron
#8	DB01119, DB01238	Diazoxide, Aripiprazole
#9	DB00564, DB01244	Carbamazepine, Bepridil
#10	DB00594, DB00422	Amiloride, Methylphenidate

## Conclusion

In this work, NMDADNN was developed to predict the interaction type of DDIs by integrating diverse drug-related information sources and combining the network-based algorithm, MDA method, DNN algorithm. The originality of NMDADNN mainly lies in that it integrates more drug-related information sources to form the drug unified feature descriptor. In the procedure of creating the drug integration features, five drug-related sources of chemical substructure information, drug–target association, drug–enzyme association, drug–pathway association, and ATC code of drugs were used to form the drug feature with the Jaccard similarity coefficient. In the process of integrating similarity matrices to generate the unified common feature descriptor, the network topological structural features of each similarity network were captured by implementing the RWR algorithm to compute PPMI values. The unified embedding features of drugs were generated by using MDA, and the DNN algorithm was adopted to predict the interaction types of DDIs. Compared with other recent state-of-the-art DNN-based methods of DeepDDI and DDIMDL, our NMDADNN method obtains the best results in terms of ACC, AUPR, AUC, F1 score, precision, and recall. The results of feature extraction and integration strategy show that capturing the network topological structural features and generating unified embedding features of drugs with MDA are the effective strategies, which improves the predictive performance.

Despite the encouraging improvement, our NMDADNN method still has the following limitations. First, NMDADNN only used five drug-related sources to generate the integration drug feature and adopted the simple similarity measure. It should be noted that more drug-related sources and suitable similarity measures can be utilized to improve the quality of drug similarity matrices. Second, DNN was used as the predictor to infer the types of DDIs; maybe another algorithm can be adopted to predict the DDIs interaction types with higher performance. Third, the number of DDIs are imbalanced for different DDIs types; thus, more techniques and parameters in NMDADNN need to optimally deal with this imbalanced data set problem. To summarize, our proposed NMDADNN is an effective approach for predicting types of DDIs. It can be expected that NMDADNN can be helpful in other type-prediction scenarios, such as the detection of side-effect types and so on.

## Data Availability

The original contributions presented in the study are included in the article/Supplementary Material, further inquiries can be directed to the corresponding author.
