# Supplementary material for: Prediction of the Drug–Drug Interaction Types with the Unified Embedding Features from Drug Similarity Networks
Source: Front Pharmacol. 2021 Dec 20;12:794205. doi: 10.3389/fphar.2021.794205 (PMC8721167; doi:10.3389/fphar.2021.794205)
Supplement: Supplementary file 1 [file DataSheet1.PDF]

Table S1 AUC scores of compared methods for each DDI-types

Types	NMDADNN	DDIMDL	DeepDDI
1	0.9427	0.9366	0.9069
2	0.9563	0.9515	0.9191
3	0.9103	0.9081	0.8816
4	0.9354	0.9235	0.9055
5	0.9310	0.9149	0.8892
6	0.9873	0.9638	0.9282
7	0.9184	0.9147	0.8283
8	0.9521	0.9464	0.9142
9	0.8742	0.8874	0.8678
10	0.9744	0.9669	0.8931
11	0.9610	0.9472	0.9274
12	0.9347	0.9413	0.9123
13	0.8952	0.7730	0.8500
14	0.9365	0.9263	0.8813
15	0.8958	0.8349	0.7919
16	0.8847	0.8816	0.8417
17	0.9938	0.9816	0.9682
18	0.9243	0.8488	0.8388
19	0.9712	0.9711	0.9364
20	0.9805	0.9935	0.9673
21	0.9641	0.9681	0.8489
22	0.9459	0.9655	0.8479
23	0.8898	0.8749	0.8296

24	0.9472	0.9367	0.9313
25	0.9510	0.8694	0.7604
26	0.9380	0.9566	0.9065
27	0.7792	0.7727	0.7269
28	0.9399	0.8932	0.8665
29	0.8785	0.8286	0.7141
30	0.9476	0.9252	0.8206
31	0.9296	0.8984	0.7964
32	0.9031	0.8466	0.8382
33	0.7117	0.7117	0.7284
34	0.9396	0.9223	0.8618
35	0.9824	0.9561	0.9473
36	0.9453	0.9363	0.8726
37	1.0000	1.0000	0.9721
38	0.8528	0.8333	0.7352
39	0.6427	0.5406	0.5610
40	0.9062	0.9166	0.8123
41	0.9886	0.9318	0.9541
42	0.9432	0.9318	0.9089
43	0.8375	0.8999	0.7874
44	0.9375	0.8625	0.8246
45	0.9625	0.9250	0.9124
46	0.8088	0.9117	0.9264
47	1.0000	0.6617	0.7645
48	0.7381	0.7381	0.5952
49	0.9500	0.9000	0.7749

50	0.6667	0.7333	0.6999
51	0.9615	0.9231	0.7692
52	0.6250	0.5416	0.4999
53	1.0000	0.6500	0.7499
54	0.8500	1.0000	0.9499
55	1.0000	0.5556	0.6666
56	0.5556	0.7778	0.6666
57	0.9444	0.9444	0.9444
58	0.6428	0.5714	0.5000
59	0.8571	0.9286	0.7143
60	1.0000	0.9167	0.7499
61	1.0000	1.0000	0.6000
62	1.0000	0.5000	0.8000
63	0.6000	0.9000	0.6000
64	0.5000	0.5000	0.5000
65	0.6999	0.6000	0.7000

Table S2 AUPR scores of compared methods for each DDI-types

Types	NMDADNN	DDIMDL	DeepDDI
1	0.9147	0.6416	0.6174
2	0.9431	0.6772	0.6331
3	0.8732	0.7152	0.6678
4	0.8835	0.8210	0.7722
5	0.9008	0.8470	0.7660
6	0.9656	0.8960	0.8263
7	0.8622	0.8274	0.6949
8	0.9071	0.8556	0.8066
9	0.7397	0.7226	0.6927
10	0.9601	0.9238	0.8290
11	0.9321	0.9092	0.8427
12	0.8483	0.8715	0.7804
13	0.7862	0.6672	0.6964

14	0.9044	0.8870	0.8128
15	0.7995	0.7809	0.6302
16	0.8389	0.8185	0.6832
17	0.9847	0.9679	0.7920
18	0.8632	0.7824	0.6500
19	0.9073	0.8767	0.8587
20	0.9739	0.9766	0.9192
21	0.9144	0.9119	0.7500
22	0.8796	0.8872	0.7634
23	0.8189	0.8087	0.6645
24	0.8768	0.8807	0.8160
25	0.8974	0.7978	0.5590
26	0.8418	0.8658	0.6554
27	0.7471	0.7595	0.5174
28	0.8922	0.8013	0.7578
29	0.8205	0.8164	0.5390
30	0.8533	0.8251	0.6502
31	0.8663	0.8443	0.5669
32	0.8132	0.7435	0.5934
33	0.5502	0.5580	0.4565
34	0.9034	0.8747	0.7050
35	0.9566	0.9362	0.8933
36	0.8608	0.8636	0.7657
37	1.0000	0.0000	0.9105
38	0.7622	0.8179	0.6093
39	0.4477	0.1512	0.1969

40	0.8397	0.8917	0.6524
41	0.9886	0.9179	0.7391
42	0.9432	0.8941	0.7754
43	0.7877	0.8315	0.6974
44	0.9376	0.8147	0.5694
45	0.9625	0.8962	0.8140
46	0.7655	0.8776	0.8175
47	1.0000	0.5049	0.5543
48	0.7382	0.6922	0.4949
49	0.9237	0.8440	0.5801
50	0.6668	0.4998	0.3872
51	0.9616	0.4998	0.6579
52	0.5001	0.2082	0.5000
53	0.9167	0.4999	0.4581
54	0.8500	0.0455	0.7712
55	1.0000	0.4999	0.4665
56	0.5557	0.6943	0.4665
57	0.8445	0.4999	0.7519
58	0.4763	0.3213	0.5000
59	0.8572	0.8570	0.5142
60	1.0000	0.4999	0.4374
61	0.9167	0.0000	0.3499
62	1.0000	0.5000	0.5499
63	0.6001	0.7999	0.3499
64	0.5001	0.4999	0.4999
65	0.3429	0.4999	0.5332

Table S3 NMDADNN predictions for DDI types unknown in DrugBank

Interaction type	DrugBank IDs	Drug names	Website
#1	DB00307, DB00745	Bexarotene, Modafinil	https://www.drugs.com/drug-interactions/bexarotene-with-modafinil-378-0-1647-0.html
#2	DB00934, DB00035	Maprotiline, Desmopressin	https://www.drugs.com/drug-interactions/desmopressin-with-maprotiline-1527-0-806-0.html
#3	DB08820, DB01204	Ivacaftor, Mitoxantrone	https://www.drugs.com/drug-interactions/ivacaftor-with-mitoxantrone-3371-0-1644-0.html
#4	DB00648, DB06413	Mitotane, Armodafinil	https://www.drugs.com/drug-interactions/armodafinil-with-mitotane-1643-0-234-0.html
#5	DB00704, DB00459	Naltrexone, Acitretin	https://www.drugs.com/drug-interactions/acitretin-with-naltrexone-1684-0-98-0.html
#6	DB00366, DB09061	Doxylamine, Cannabidiol	https://www.drugs.com/drug-interactions/cannabidiol-with-doxylamine-942-0-3919-0.html
#7	DB00537, DB00969	Ciprofloxacin, Alosetron	https://www.drugs.com/drug-interactions/alosetron-with-ciprofloxacin-672-0-130-0.html
#8	DB01119, DB01238	Diazoxide, Aripiprazole	https://www.drugs.com/drug-interactions/aripiprazole-with-diazoxide-863-0-233-0.html
#9	DB00564, DB01244	Carbamazepine, Bepridil	https://www.drugs.com/drug-interactions/bepridil-with-carbamazepine-497-0-366-0.html
#10	DB00594, DB00422	Amiloride, Methylphenidate	https://www.drugs.com/drug-interactions/amiloride-with-methylphenidate-154-0-1606-0.html
